# Enhancing/Improving Forming Limit Curve and Fracture Height Predictions in the Single-Point Incremental Forming of Al1050 Sheet Material

**DOI:** 10.3390/ma16237266

**Published:** 2023-11-21

**Authors:** Trung-Kien Hoang, The-Thanh Luyen, Duc-Toan Nguyen

**Affiliations:** 1 Faculty of Mechanical Engineering, Thainguyen University of Technology, Thainguyen 250000, Vietnam; htk@tnut.edu.vn; 2 Faculty of Mechanical Engineering, Hungyen University of Technology and Education, Hungyen 160000, Vietnam; luyenthethanh@gmail.com; 3 School of Mechanical Engineering, Hanoi University of Science and Technology, 1A-Dai Co Viet Street, Hai Ba Trung District, Hanoi City 100000, Vietnam

**Keywords:** SPIF (single-point incremental forming), forming limit curve at fracture (FLCF), forming limit curve at necking (FLCN), modified maximum force criterion (MMFC), graphical method, sheet material Al1050, Finite Element Method (FEM)

## Abstract

Single-point incremental forming (SPIF) has emerged as a cost-effective and rapid manufacturing method, especially suitable for small-batch production due to its minimal reliance on molds, swift production, and affordability. Nonetheless, SPIF’s effectiveness is closely tied to the specific characteristics of the employed sheet materials and the intricacies of the desired shapes. Immediate experimentation with SPIF often leads to numerous product defects. Therefore, the pre-emptive use of numerical simulations to predict these defects is of paramount importance. In this study, we focus on the critical role of the forming limit curve (FLC) in SPIF simulations, specifically in anticipating product fractures. To facilitate this, we first construct the forming limit curve for Al1050 sheet material, leveraging the modified maximum force criterion (MMFC). This criterion, well-established in the field, derives FLCs based on the theory of hardening laws in sheet metal yield curves. In conjunction with the MMFC, we introduce a graphical approach that simplifies the prediction of forming limit curves at fracture (FLCF). Within the context of the SPIF method, FLCF is established through both uniaxial tensile deformation (U.T) and simultaneous uniform tensile deformation in bi-axial tensile (B.T). Subsequently, the FLCF predictions are applied in simulations and experiments focused on forming truncated cone parts. Notably, a substantial deviation in fracture height, amounting to 15.97%, is observed between simulated and experimental samples. To enhance FLCF prediction accuracy in SPIF, we propose a novel method based on simulations of truncated cone parts with variable tool radii. A FLCF is then constructed by determining major/minor strains in simulated samples. To ascertain the validity of this enhanced FLCF model, our study includes simulations and tests of truncated cone samples with varying wall angles, revealing a substantial alignment in fracture height between corresponding samples. This research contributes to the advancement of SPIF by enhancing our ability to predict and mitigate product defects, ultimately expanding the applicability of SPIF in diverse industrial contexts.

## 1. Introduction

Single-point incremental forming (SPIF) has gained prominence as a cost-effective and rapid method for manufacturing sheet metal parts, complementing traditional machining techniques, and seamlessly integrating with computer numerical control (CNC) machines. Its versatility has led to its adoption across a multitude of industries, including aerospace [1,2], automotive [3,4], molds [5], medical devices [6,7,8], architecture [9,10], and beyond. Nevertheless, challenges persist in SPIF, particularly regarding defects in wall thickness uniformity, geometric accuracy, and other process limitations, which impede its broader industrial applications. To address these limitations and enhance SPIF’s predictability, a considerable body of research has been dedicated to numerical simulations of the forming process [11,12,13,14,15,16,17].

Bouhamed et al. [18] have concentrated on advancing the accuracy of SPIF simulations by introducing material anisotropy considerations, employing Hill’s models to achieve improved fits with experimental data. Han et al. [19] have extended their investigations to establish forming limit curves at fracture (FLCF) for various sheet materials, employing plane stress conditions and Barlat’s non-quadratic anisotropic yield criterion. Their comprehensive approach, which combines the Cockroft–Latham ductile fracture criterion and maximum shear stress criteria, has yielded a means to accurately predict FLCFs with diverse shapes. In a parallel line of inquiry, Rusu et al. [20] study the SPIF behavior of Al1050 material sheets with differing thicknesses. This material was joined through both one-sided and two-sided Wolfram inert gas (WIG) welding methods, resulting in truncated cone profiles with varying wall angles. Their findings revealed significant differences in tear behavior, with double-sided welding achieving the desired depth of 25 mm, while single-sided welding often resulted in premature tearing. Xiao et al. [21] delved into the influence of longitudinal low-frequency vibrations on the performance of SPIF with AA1050 aluminum sheet material. Their study integrated numerical simulations and experimental verification to showcase the significant reduction in forming forces and improvement in geometric accuracy through the application of low-frequency vibrations.

Shang et al. [22] explored the use of hydraulic oil pressure to enhance thickness uniformity and critical forming angles in hydraulically supported single-point incremental forming (HS-SPIF). Through finite element simulations and experimental comparisons, they determined the sensitivity of critical forming angles to hydraulic pressure. The research highlighted substantial differences between conventional SPIF and HS-SPIF, with critical angles ranging from 47 to 53°.

Saidi et al. [17] focused on the SPIF of Ti–6Al–4V sheet material, relevant in the production of medical prosthetics. By incorporating heating during the machining process, they significantly improved formability. The study introduced a simulation model at different temperatures, providing accurate predictions compared to corresponding experimental outcomes.

In this study, the modified maximum force criterion (MMFC) was harnessed to predict the forming limit curve of Al1050 sheet material. A graphical method was devised to simplify FLC forecasting, constructed through uniaxial tensile deformation (U.T) and simultaneous biaxial tensile deformation (B.T). The deviation in fracture height between the simulated and experimental samples was notably large, reaching 15.97%. To enhance FLCF in SPIF, this study proposes a method based on simulations of truncated cone parts with varying tool radii and the determination of major and minor strains in simulated samples. The verification of the improved FLCF model involved simulating and testing truncated cone samples with different wall angles, demonstrating a significant agreement in fracture height between corresponding samples. This research endeavors to contribute to the further development and applicability of SPIF across a wide array of industrial domains.

## 2. Material Properties

This section delves into an extensive examination of the material properties that are integral to this study’s focus on Al1050 aluminum alloy sheets with a thickness of 0.5 mm. This material was chosen as a fundamental element in the investigation, and understanding its chemical composition and mechanical characteristics is pivotal in comprehending its performance in the context of SPIF of truncated cone parts. The chemical composition of the Al1050 aluminum alloy, as referenced in [23], assumes a pivotal role in shaping its properties, necessitating a detailed investigation.

**Figure 1:**
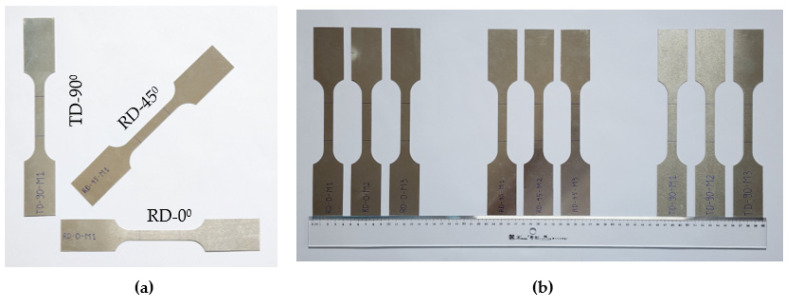
Tensile test samples of Al1050 aluminum alloy material: (**a**) cutting direction of tensile test specimens in rolling direction (RD)—transverse direction (TD) and (**b**) material samples cut in three directions.

Material anisotropy, an aspect profoundly impacting the quality of the fabricated product, was a significant focus of this research. Tensile test samples were prepared via waterjet cutting in three specific directions: the rolling direction (RD), the horizontal direction perpendicular to the rolling direction (TD), and a direction 45 degrees from the rolling direction. As demonstrated in Figure 1a,b, these directions were chosen strategically for their relevance.

**Figure 2:**
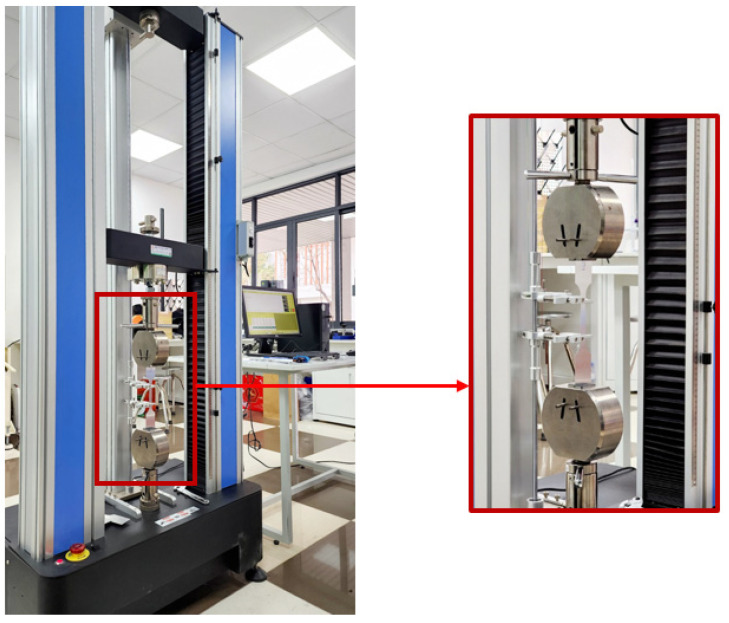
Tensile test performed on YS-L45D1-J11 machine.

**Figure 3:**
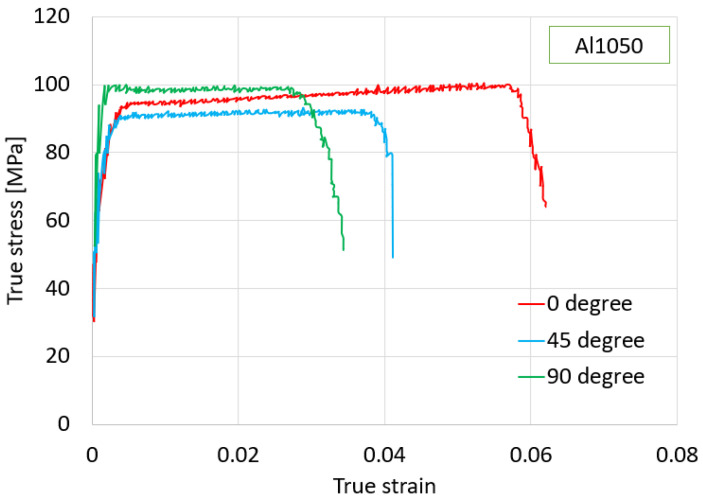
True stress–true strain curves of Al1050 material in three directions: rolling direction, (45°), and transverse direction (90°).

Subsequent tensile tests were conducted at room temperature on a YS-L45-J11 machine (Dongguan, China), as depicted in Figure 2. These tests adhered to ISO 6892-1 standards [24], providing consistent and reliable data for analysis. The results of these tensile tests encompass the stress–strain curves, presented graphically in Figure 3, and are condensed into a concise summary of material properties presented in Table 1.

**Figure 4:**
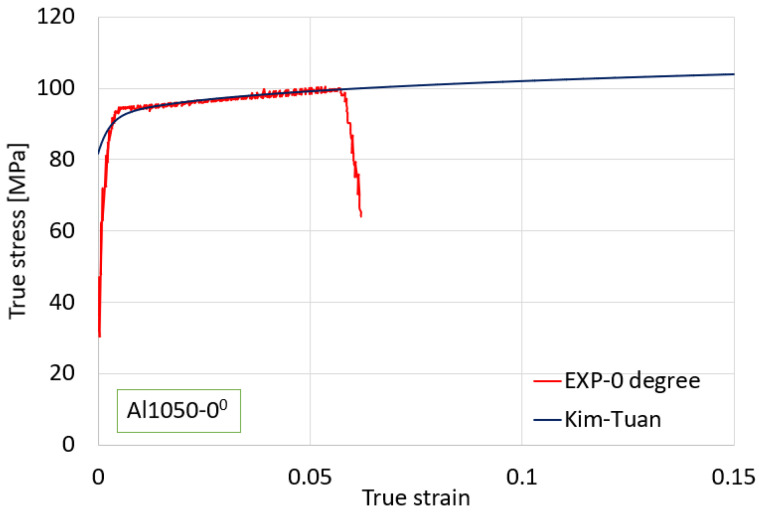
Kim–Tuan Model and curve in the RD.

The investigation aimed to determine an appropriate plasticity model for the material. The stress–strain curve of Al1050 in the rolling direction (RD), as presented in Figure 4, was utilized for this purpose. The chosen plasticity model is the Kim–Tuan model, which is well detailed in Equation (1). To quantify the strain hardening behavior, several strain hardening equations are available in the literature. In this study, we followed the approach outlined in references [25,26,27]. Notably, we adopted the Kim–Tuan strain hardening model, which has demonstrated a strong fit and high accuracy in modeling strain hardening behavior. The choice of the Kim–Tuan model was made after a careful consideration of its appropriateness for our material and the excellent correlation between predictions and experimental results:

(1) $$\bar{\sigma }=\sigma_{0}+K(1-\exp(-t\bar{\varepsilon })){\left(\bar{\varepsilon }+0.002\right)}^{h}$$

where $\bar{\sigma }$ represents the true stress. $\bar{\varepsilon }$ represents the true strain. *σ_Y_* stands for the yield strength (81.6 MPa). *K*, *t*, and *h* are model coefficients, with values of *K* = 34.26 MPa, *t* = 471.62, and *h* = 0.225. These coefficients were meticulously determined through Formulas (2a)–(2c), with a deviation threshold of 0.2%.

Formulas for coefficients (2a)–(2c):

(2a) $$h=\frac{\sigma^{*}}{\sigma^{*}-\sigma_{0}}(\varepsilon^{*}-\varepsilon_{0})$$

(2b) $$K=\frac{\sigma^{*}-\sigma_{0}}{(\varepsilon^{*}-\varepsilon_{0}{)}^{h}}$$

(2c) $$t=\frac{20}{\varepsilon^{*}}$$

In this research, the Hill’48R stress function, detailed in Equation (3), is employed to deduce the anisotropy parameters governing the material’s behavior.

General equation for equivalent stress (Equation (3)):

(3) $${\bar{\sigma }}^{2}=H(\sigma_{11}-\sigma_{22}{)}^{2}+F(\sigma_{22}-\sigma_{33}{)}^{2}+G(\sigma_{33}-\sigma_{11}{)}^{2}+2L\sigma_{23}^{2}+2M\sigma_{31}^{2}+2N\sigma_{12}^{2}$$

$\bar{\sigma }$: equivalent stress. $\sigma_{11},\sigma_{22},\sigma_{33},\sigma_{11},\sigma_{23},$ and $\sigma_{31}:$ stress components determined in various directions. *G*, *F*, *H*, *N*, *L*, *M*: material constants requiring determination based on experimental data.

In the case of plane stress conditions, Equation (3) can be simplified as Equation (4).

(4) $$\bar{\sigma }=\sqrt{H(\sigma_{11}-\sigma_{22}{)}^{2}+F\sigma_{22}^{2}+G\sigma_{11}^{2}+2N\sigma_{12}^{2}}$$

For this study, anisotropy values (*r*_0_, *r*_90_, *r*_45_) were derived from three uniaxial tensile tests (0°, 45°, and 90° with respect to the RD). Additionally, the uniaxial yield stress (*σ*₀) was obtained, maintaining a consistent relationship (*G* + *H* = 1). Recognizing the difficulty of evaluating anisotropy in the direction of material thickness, the properties were assumed to be isotropic (*L* = *M* = 1.5).

The Hill’48R stress criterion, under plane stress conditions ($\sigma_{33}=\sigma_{23}=\sigma_{31}=0$), was instrumental in predicting the uniaxial yield stress in direction *θ*, as expressed in Equation (5).

(5) $$\sigma_{\theta }=\frac{\bar{\sigma }}{\sqrt{(F+H)\sin^{4}\theta +(G+H)\cos^{4}\theta +2(N-H)\sin^{2}\theta \cos^{2}\theta }}$$

Subsequently, uniaxial anisotropy in the same rolling direction (*θ*) was determined utilizing the stress criterion in Equation (6).

(6) $$r_{\theta }=\frac{H+(2N-4H-G-F)\sin^{2}\theta \cos^{2}\theta }{F\sin^{2}\theta +G\cos^{2}\theta }$$

The anisotropy parameters for the Hill’48R stress criterion [28] were established based on the experimental yield stress and the coefficient of plastic anisotropy, as presented in Table 1. Meanwhile, Table 2 encapsulates the parameters of the Hill’48R yield criterion, with coefficients calculated following Equations (7a)–(7d).

(7a) $$G=\frac{1}{1+R_{0}}$$

(7b) $$H=\frac{R_{0}}{1+R_{0}}$$

(7c) $$F=\frac{R_{0}}{R_{90}(1+R_{0})}$$

(7d) $$N=\frac{\left(R_{0}+R_{90})(1+2R_{45}\right)}{2R_{90}(1+R_{0})}$$

The subsequent calculation of anisotropy coefficients (*R*_11_, *R*_22_, *R*_33_, *R*_12_, *R*_13_, *R*_23_) from these coefficients (Table 2) is detailed in Equation (8), and the outcomes are displayed in Table 3.

Formulas for coefficients (8):

(8) $$\left\{\begin{array}{l} R_{11}=\sqrt{\frac{1}{G+H}}\\ R_{22}=\sqrt{\frac{1}{F+H}}\\ R_{33}=\sqrt{\frac{1}{F+G}}\\ R_{23}=\sqrt{\frac{3}{2L}},R_{13}=\sqrt{\frac{3}{2M}},R_{12}=\sqrt{\frac{3}{2N}} \end{array}\right.$$

**Figure 5:**
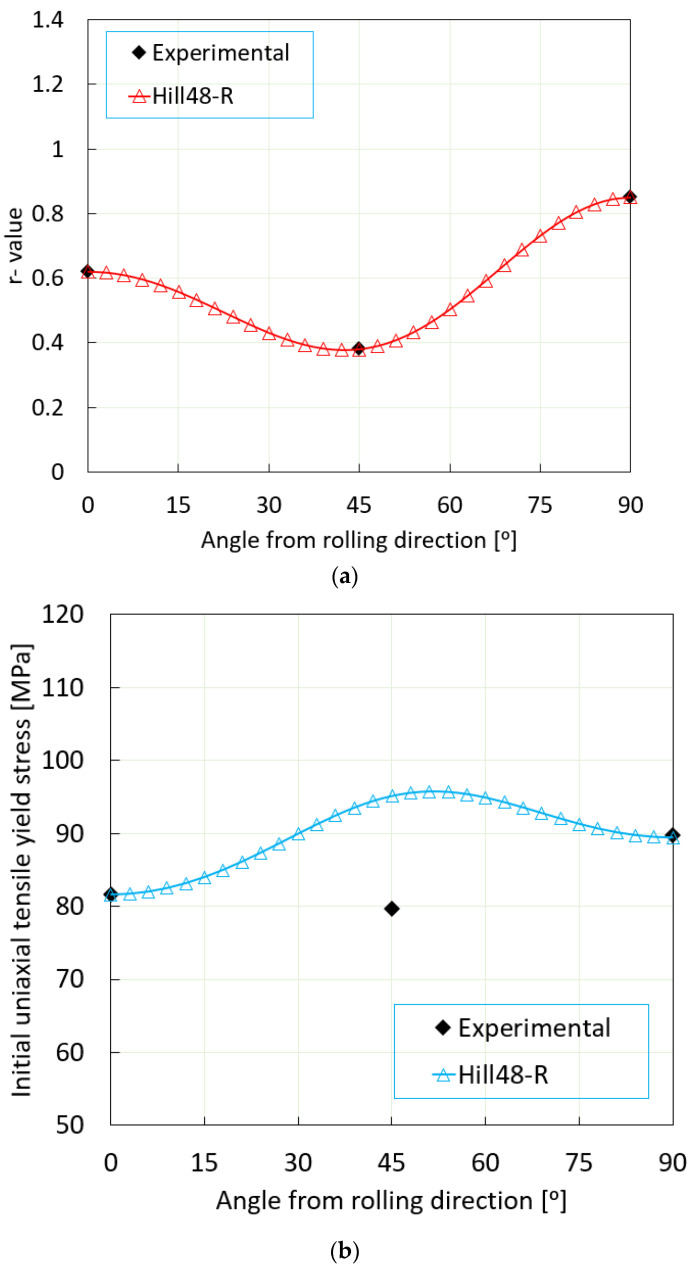
Comparison of anisotropy coefficient between model and experiment: (**a**) uniaxial tensile anisotropy coefficient and (**b**) different stress criteria.

A comprehensive investigation was conducted to examine the anisotropy coefficient’s alignment with the Hill’48R stress criteria against experimental data. This comparative analysis, as demonstrated in Figure 5a,b, brings to light the distribution of in-plane uniaxial anisotropy coefficients and stress distributions in different directions relative to the rolling direction. The apparent variance in yield stress for the 45° rolling direction between the model and experimental data is likely attributable to the particular values of these coefficients and the influence of the anisotropy model in this specific orientation (Figure 5b). It is crucial to recognize that anisotropic models can provide precise predictions that are contingent upon the specific criteria employed. Further research efforts are imperative to delve into the intricacies of anisotropic modeling and its ramifications on predictive accuracy. This is especially pertinent for the single-point incremental forming (SPIF) method, which warrants more in-depth exploration in future investigations.

**Figure 6:**
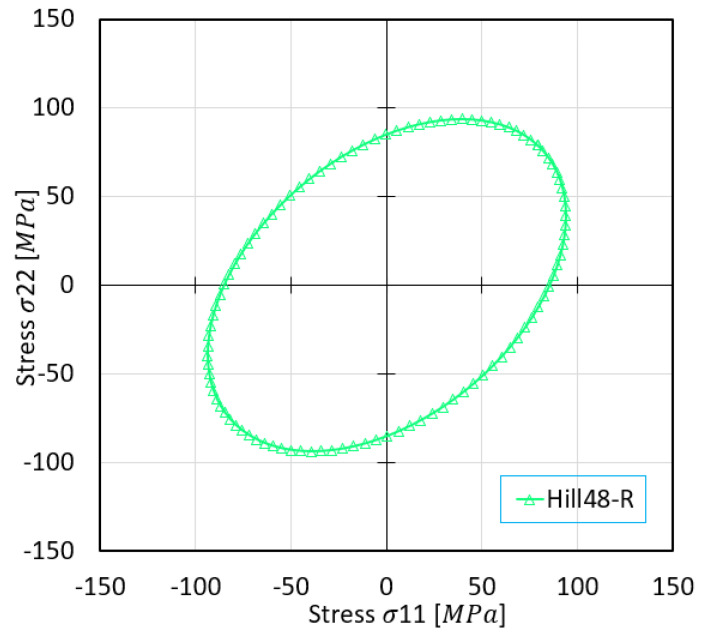
Stress surface predicted based on Hill’48R criteria.

The predicted stress surfaces were plotted using varying yield criteria for *σ*_11_ and *σ*_22_, as showcased in Figure 6, ultimately yielding a maximum stress value based on the Hill’48R stress model.

This comprehensive exploration of material properties paves the way for a deep understanding of Al1050 aluminum alloys and their mechanical characteristics, enabling informed and rigorous numerical simulations and experiments. This research builds on these foundations to draw insightful conclusions and offer innovative contributions to the field.

## 3. Prediction of the Forming Limit Curve (FLC)

### 3.1. Modified Maximum Force Criterion Method

In the context of uniaxial tensile testing, the determination of the maximum tensile force can be formulated through Equation (9):

(9) dF=d(σS)=Sdσ+σdS=0

Here, *S* denotes the cross-sectional area of the test specimen and *F* represents the measured tensile force. The condition is further expressed as

(10) $$\sigma =-d\sigma \frac{S}{dS}=\frac{d\sigma }{d\varepsilon }$$

Upon careful observation, Swift [29] introduced a theory of crack propagation and progression criteria to estimate the plastic deformation limit within the forming limit curve (FLC) for sheet metals. This criterion has gained widespread recognition for predicting the FLC of diverse materials. Subsequently, Hora et al. [30] introduced a criterion termed the modified maximum force criterion (MMFC), which involves the examination of strain path transitions following the appearance of cracks. The expression for the MMFC is articulated as

(11) $$\frac{\partial \sigma_{1}}{\partial \varepsilon_{1}}d\varepsilon_{1}+\frac{\partial \sigma_{1}}{\partial \beta }\frac{\partial \beta }{\partial \varepsilon_{1}}d\varepsilon_{1}\geq \sigma_{1}d\varepsilon_{1},$$

Here, $\beta =\Delta \varepsilon_{2}/\Delta \varepsilon_{1}$ represents the deformation ratio in two primary directions and $\alpha =\sigma_{2}/\sigma_{1}$ signifies the stress ratio in the two principal directions. Two functions, $f(\alpha )=\bar{\sigma }/\sigma_{1}$ and $g(\alpha )=\Delta \bar{\varepsilon }/\Delta \varepsilon_{1}$, depict the relationship between the first principal stress ($\sigma_{1}$), the strain ($\varepsilon_{1}$) components, and the ($\bar{\sigma }$) and strain ($\bar{\varepsilon }$) values, respectively. For each chosen hardening function within the stress–strain relationship, the terms $\partial \sigma_{1}/\partial \varepsilon_{1}$ and $\partial \sigma_{1}/\partial \beta_{1}$ can be expressed as follows:

(12) $$\frac{\partial \sigma_{1}}{\partial \varepsilon_{1}}=\frac{\partial \sigma_{1}}{\partial \bar{\sigma }}\frac{\partial \bar{\sigma }}{\partial \bar{\varepsilon }}\frac{\partial \bar{\varepsilon }}{\partial \varepsilon_{1}}=g(\alpha )H^{\prime }/f(\alpha )$$

(13) $$\frac{\partial \sigma_{1}}{\partial \beta }=\frac{\partial \sigma_{1}}{\partial \alpha }\frac{\partial \alpha }{\partial \beta }=-\frac{f^{\prime }(\alpha )}{[f(\alpha ){]}^{2}}\bar{\sigma }\frac{\partial \alpha }{\partial \beta }=-\frac{f^{\prime }(\alpha )}{[f(\alpha ){]}^{2}}H/(\frac{\partial \beta }{\partial \alpha })$$

In Equations (12) and (13), $H=H(\bar{\varepsilon })$ represents the hardening function, and *H*’ represents the slope of the hardening curve. According to the principles of continuum mechanics, we can establish the following:

(14) $$\beta =\frac{d\varepsilon_{2}}{d\varepsilon_{1}}=\frac{\partial \bar{\sigma }/\partial \sigma_{2}}{\partial \bar{\sigma }/\partial \sigma_{1}}$$

Consequently, β'(α) becomes precisely defined. The evaluation of $\partial \beta /\partial \varepsilon_{1}$ necessitates an iterative approach. Nevertheless, to enhance efficiency and simplify calculations, $\beta \approx \varepsilon_{2}/\varepsilon_{1}$ is adopted as an approximation. Therefore, the derivative obtained is

(15) $$\frac{\partial \beta }{\partial \varepsilon_{1}}\approx -\frac{\beta }{\varepsilon_{1}}$$

Substituting Equations (12)–(15) into Equation (11) yields an explicit formulation of the modified maximum force criterion (MMFC) as

(16) $$\frac{H^{\prime }}{H}\geq \frac{1}{g(\alpha )}\left[1-\frac{f^{\prime }(\alpha )}{f(\alpha )}\frac{\beta }{\beta^{\prime }(\alpha )}\frac{1}{\varepsilon_{1}}\right]$$

### 3.2. Graphical Method for Al1050 Material

In this study, a graphical method was utilized to construct the forming limit curve (FLC) for an Al1050 aluminum alloy sheet. This approach encompasses specific deformation modes, including the plane strain (P.S) (*β* = 0), uniaxial tensile strain (U.T) (*β* = −1/2), and uniform tensile strain in bi-axial tensile (B.T) (*β* = 1), which are commonly applied in traditional forming methods. For the single-point incremental forming (SPIF) method, the FLC is determined as discussed by various authors [31,32] In this context, both FLCN and FLCF for equi-biaxial strain conditions nearly converge at a single point and exhibit linear behavior as they pass through two deformation states, characterized by *β* values of −1/2 and 1. This method is explicitly defined in accordance with Equation (16) and can be subsequently transformed into Equation (17a).

(17a) $$\frac{H'}{H}\geq A-\frac{B}{\varepsilon }$$

(17b) A=1/g(α)

(17c) B=(f'/f)×(β/β')

The values of *A*(*α*) and *B*(*α*) can be determined from the coefficients of the Hill’48R stress function, calculated as per Equations (18a)–(19b). Specific deformation modes such as the uniaxial tensile strain (U.T) (β=−1/2) and simultaneous bi-axial uniform tensile strain (B.T) (β=1) are included in Table 4.

**Figure 7:**
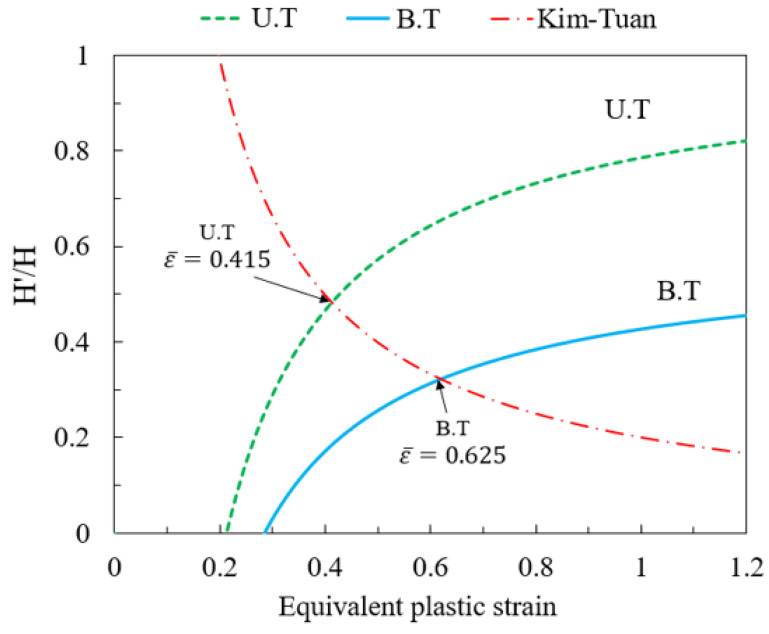
Graphical method for predicting the forming limit curve of Al1050 sheet metal.

Based on the computed values and the previously mentioned equations, Equation (17a) can be graphically represented as illustrated in Figure 7, employing the Kim–Tuan models.

(18a) $$f(\alpha )=\sqrt{(G+H)-2H\alpha +(F+H)\alpha^{2}}$$

(18b) $$f^{\prime }(\alpha )=\frac{\left[-H+(F+H)\alpha \right]}{\sqrt{(G+H)-2H\alpha +(F+H)\alpha^{2}}}$$

(18c) $$\beta (\alpha )=\frac{(F+H)\alpha -H}{-H\alpha +(G+H)}$$

(18d) $$\beta^{\prime }(\alpha )\frac{(F+H)(G+H)-H^{2}}{[-H\alpha +(G+H){]}^{2}}$$

(18e) $$g(\alpha )=\frac{\sqrt{(G+H)-2H\alpha +(F+H)\alpha^{2}}}{-H\alpha +(G+H)}$$

Subsequently,

(19a) $$A(\alpha )=\frac{-H\alpha +(G+H)}{\sqrt{(G+H)-2H\alpha +(F+H)\alpha^{2}}}$$

(19b) $$B(\alpha )=\frac{-H+(F+H)\alpha }{(G+H)-2H\alpha +(F+H)\alpha^{2}}\times \frac{\left[(F+H)\alpha -H]\times [-H\alpha +(G+H)\right]}{(F+H)(G+H)-H^{2}}$$

**Figure 8:**
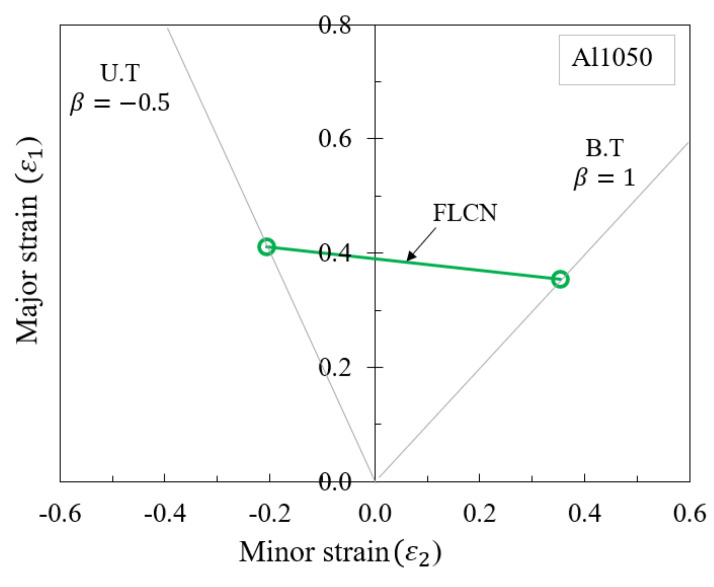
Forming limit curve (FLC) determination using the graphical method for Al1050 sheet material.

The graphical method, as demonstrated in Figure 7, reveals the intersection of equivalent destructive deformation lines (U.T) and (B.T) with the curve constructed based on the hardening model, utilizing corresponding values of $\bar{\varepsilon }$ = 0.415 and $\bar{\varepsilon }$ = 0.625. Subsequently, the major and minor strains are determined using Equations (20a)–(20c). The forming limit curve for the Al1050 aluminum alloy sheet can be obtained by plotting from these two distinct deformation points, as outlined in Table 5 and depicted in Figure 8.

(20a) $$\varepsilon_{1}=\bar{\varepsilon }/\frac{r_{m}+1}{\sqrt{2r_{m}+1}}\sqrt{1+\frac{2r_{m}}{r_{m}+1}\beta +\beta^{2}}$$

(20b) $$\varepsilon_{2}=\beta \varepsilon_{1}$$

(20c) $$r_{m}=\frac{1}{4}(r_{0}+2r_{45}+r_{90})$$

## 4. Experiment and Finite Element (FE) Simulation

### 4.1. Finite Element Simulation

**Figure 9:**
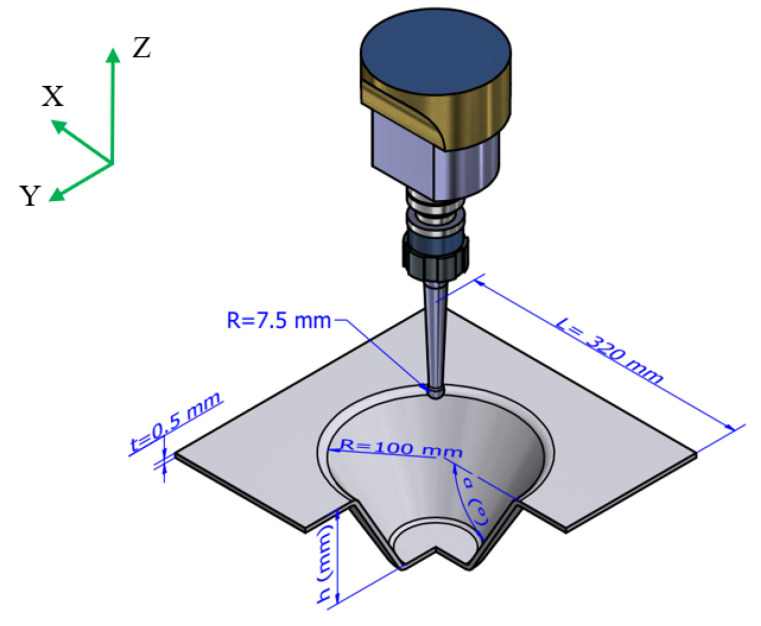
Parameters of the SPIF machining model.

For the finite element simulations, the Al1050 aluminum material was employed. The material took the form of a square with a side length (L) of 320 mm and a thickness (t) of 0.5 mm. The forming tool used in these simulations had a spherical radius of 7.5 mm. The desired forming profile was that of a truncated cone, with a base radius (R) of 100 mm. The forming height was denoted as “h” (in millimeters), and the forming angle as “α” (in degrees). These parameters are modeled in Figure 9.

**Figure 10:**
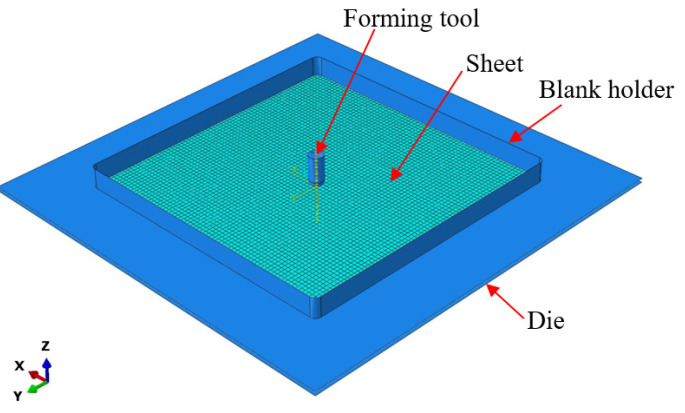
SPIF model in ABAQUS 6.13 software.

**Figure 11:**
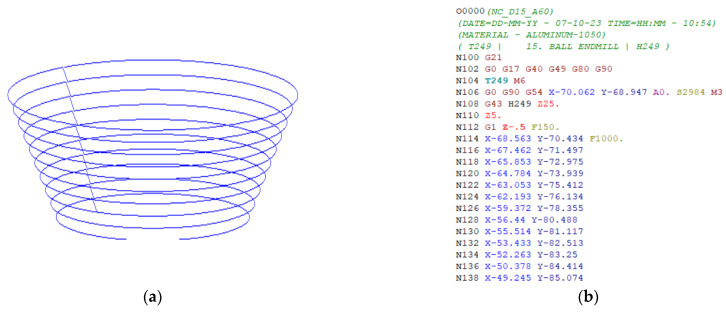
Toolpath used in numerical simulation (**a**); Numerical control (NC) code used in practice (**b**).

The finite element model was established to replicate the single-point incremental forming (SPIF) process, using the ABAQUS 6.13 software [33]. As illustrated in Figure 10, the FE model incorporated the following components: an Al1050 aluminum alloy sheet, the forming tool, a sheet support die plate, and a blank holder. The aluminum alloy sheet was represented as a shell element with an integrated number of reduction points (S4R) at a mesh size of approximately 1 mm in width and 1 mm in length. On the other hand, the tool, support die plate, and blank holder were treated as entirely rigid. Fixed boundary conditions were assigned to the support die and blank holder, while the aluminum alloy sheet was clamped firmly and subjected to the vertical downward force applied by the tool. The toolpath of the SPIF process, outlined in Figure 11a, matched the code employed in the experimental setup. Friction within the system was defined using the classical isotropic Coulomb friction model, governed by the contact surface coefficient friction force. Specifically, the friction coefficient between the tool and the sheet blank was set to 0.05, while the friction coefficient between the die, stop plate, and the sheet blank was established as 0.1 [31]. The material parameters are outlined in Table 1, and the anisotropy coefficients of the material, along with the FLC, were determined using the graphical method during the numerical simulation process. This information was then utilized to calculate the height of the forming for the truncated cone part in the numerical simulation.

### 4.2. SPIF Experiment

The SPIF experiments for the truncated cone samples were carried out on a Taiwanese MC500 high-speed CNC machine (Fuhong Machinery Co., New Taipei, Taiwan). The machine’s table offered movement along the X × Y × Z axes of 500 mm × 400 mm × 300 mm, with a spindle rotation speed ranging from 100 to 30,000 rpm, table movement speeds during machining from 1 to 30,000 m/min, and a spindle power rated at 15 kW. The numerical control (NC) toolpath was generated using the PowerMill 2021 software, based on the computer-aided design (CAD) geometry of the machined sample, with the offset code corresponding to Figure 11b. In these experiments, the spindle rotation speed was set to S = 2984 rpm, the feed step (F) was 1000 mm/min, and the depth of cut (t) was 0.5 mm.

**Figure 12:**
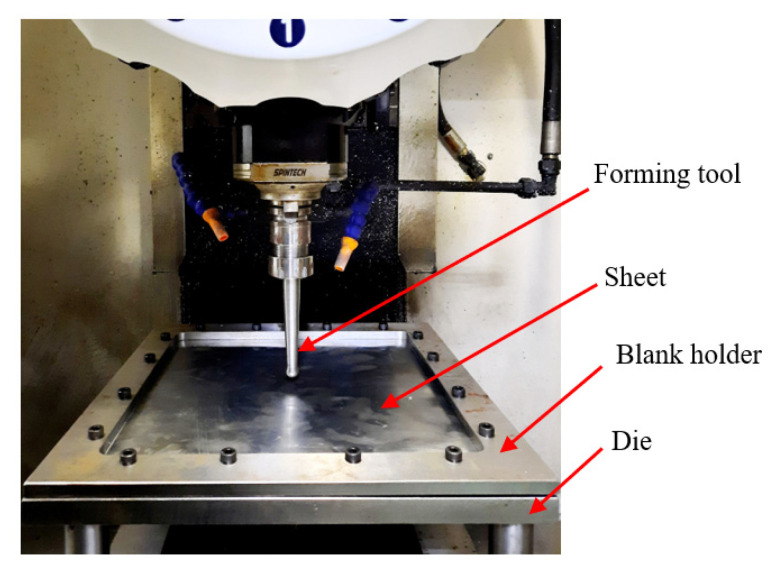
Experimental setup for the incremental forming process.

The experimental setup for the SPIF process is shown in Figure 12. The blank was securely clamped between the fixed support die plate and the blank holder, using a 10 mm diameter bolt. The fixed support die plate was crafted from High-Carbon High-Chromium alloy steel (SKD11) material, while other fixture components were made from medium carbon steel (C45) material. Viscosity grade-68 (VG68) hydraulic oil was employed as the lubricant during the experiment to minimize surface friction between the tool and the blank. The forming tool had a spherical tip with a diameter of 15 mm and was constructed from the SKD11 material, hardened to a hardness of 55HRC. The smoothness of the tool at the spherical tip position was measured at Ra = 0.25 µm. The Al1050 aluminum alloy plate utilized in the experiment had a thickness of 0.5 mm.

## 5. Results and Discussion

### 5.1. Comparison of Fracture Height in SPIF Simulation and Experiment

**Figure 13:**
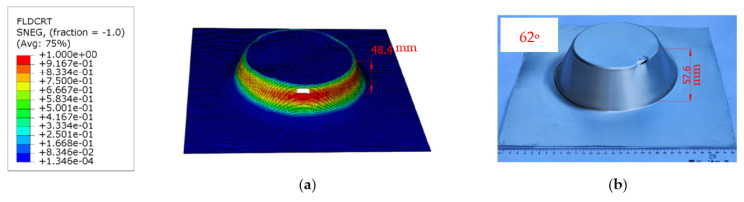
Comparison of fracture height in the SPIF simulation (**a**) and experiment (**b**).

In the simulations, the damage evolution criterion is a crucial component used to model material failure and fracture. It is defined as the point at which the forming limit curve ductile fracture (FLDCRT) value reaches 1.0, which indicates a fracture condition. When this criterion is met, certain mesh elements within the simulation are deleted, and the maximum fracture height is recorded for the corresponding wall corners. The comparison between the detailed fracture height in the SPIF simulation and the experimental results is presented in Figure 13. The deviation between these values is calculated as a percentage difference using Equation (21). The results are summarized in Table 6.

The finite element simulation, employing the Kim–Tuan hardening law in the RD, along with the anisotropic Hill’48R model and the FLC constructed through graphical methods, aimed to predict the increase in fracture height during forming processes. As shown in Figure 13a,b, both the simulated and experimental images of truncated cone details are compared. The fracture heights and deviations between the two are detailed in Table 6, showing a deviation of 15.97%. This considerable deviation emphasizes the need for a new approach to predict the FLC in SPIF under varying tool radii.

(21) $$\Delta h(\%)=(\left|h_{s}-h_{e}\right|)/h_{e}$$

### 5.2. Proposed Forming Limit Curves at Fracture (FLCF) in SPIF

In SPIF, forming limit curves (FLCs) serve as useful tools for simulating forming and bending processes, especially when material formability is limited under increased forming conditions. Previous studies have demonstrated that when bending and forming loads are applied simultaneously, the formability of materials can exceed the strain values expected from typical FLC predictions. Notably, the FLCs in progressive sheet forming (FLCN for necking and FLCF for fracture) exhibit a near convergence at isotropic strain. Consequently, this research leveraged Clift’s criterion [34] to propose a new point on the FLCF, using the major/minor strains derived from Equation (20b) and the equivalent strain function of plane stress from Equation (20a). The constant value for this FLCF, represented as the equivalent strain at fracture (${\bar{\varepsilon }}_{f})$, is determined using Equations (22) and (23).

(22) $$\int_{0}^{\bar{\varepsilon }}\bar{\sigma }d\bar{\varepsilon }=C$$

(23) $${\bar{\varepsilon }}_{f}=C_{1}$$

where *C* and $C_{1}$ are material parameters.

To calculate $C_{1}$, data from the strain ratio and the principal strain at the isoaxial point were utilized. The calculations for $C_{1}$ were performed using an algorithm developed by Shi et al. [35] and Son et al. [36]. In biaxial equilibrium conditions, the deformation coefficient (*β*) was set at 1.0. Additionally, the principal fracture strain for a tool radius (R) of 7.5 mm was found to be 0.452, yielding a constant value ($C_{1}$) of 0.798. Consequently, multiple points on the FLCF were derived using various strain values, and the values were computed with the help of MATLAB programming.

**Figure 14:**
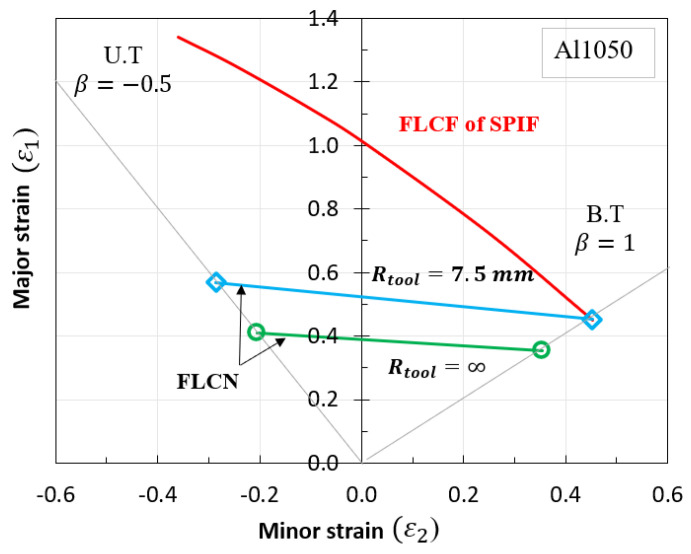
Proposed FLCF of Al1050 material.

**Figure 15:**
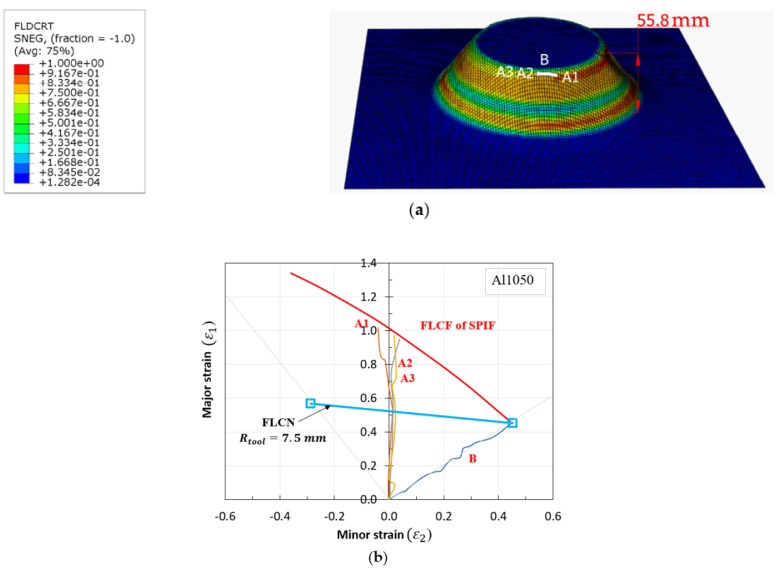
Deformed shape with proposed FLCF (**a**) and strain path from FEM (**b**).

Data from the MATLAB calculations (Figure 14) were exported into two columns corresponding to major (ε_1_) and minor (ε_2_) strains. These columns were subsequently integrated into the FLC damage table within the material properties module of ABAQUS/Explicit to simulate the fracture occurrence in incremental sheet metal forming processes. The finite element (FE) simulation results of the SPIF process with the proposed FLCF are visualized in Figure 15a. The strain paths at different fracture points of the deformed shape are also illustrated, and a comparison of the forming limit curve for fracture (FLCF) is presented in Figure 15b, demonstrating a high level of agreement between the simulation and the proposed FLCF.

### 5.3. Verification of the Proposed FLCF with Varying Forming Angles

**Figure 16:**
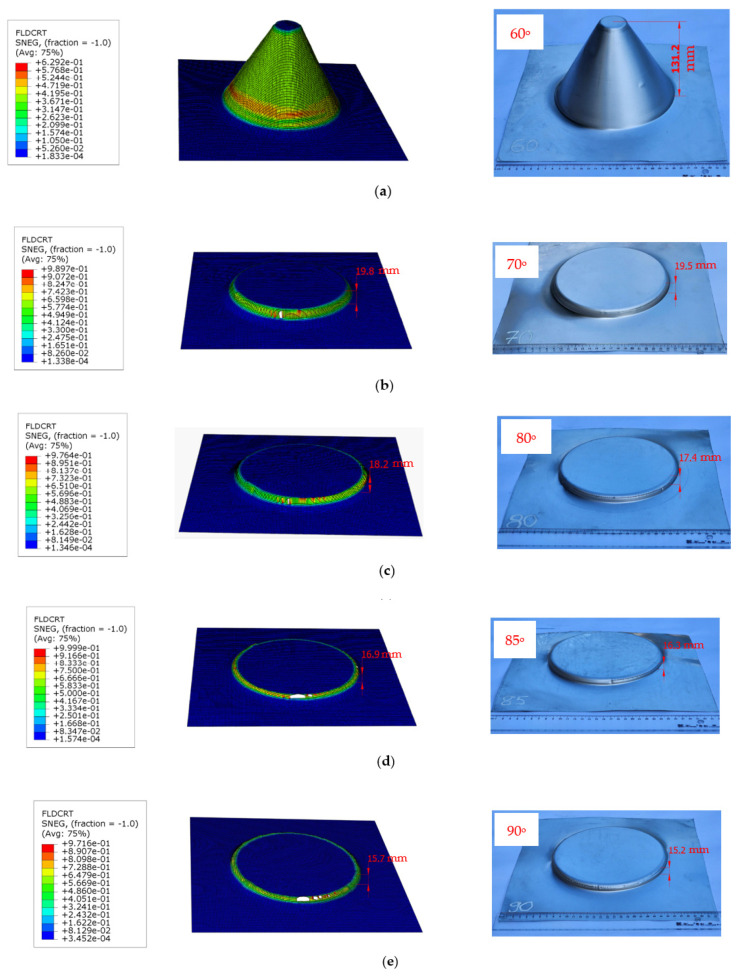
Height of forming a truncated cone detail for various forming wall angles: (**a**) 60°, (**b**) 70°, (**c**) 80°, (**d**) 85°, and (**e**) 90°.

The reliability of the proposed FLCF was verified by the predicting failure occurrences and fracture heights for samples with varying forming angles. The finite element (FE) simulation results for these experimental samples are displayed in Figure 16 and summarized in Table 7.

**Figure 17:**
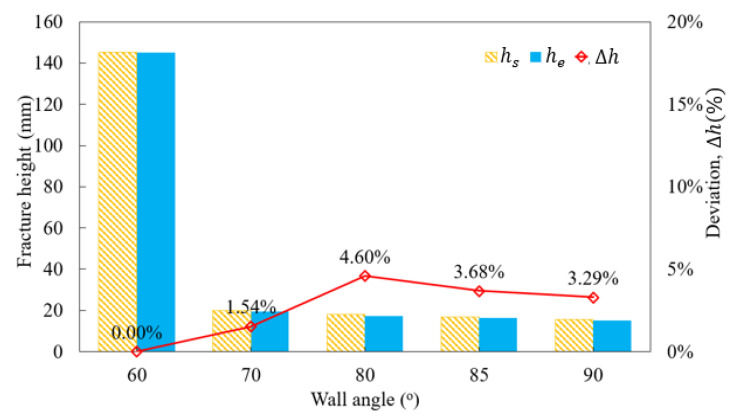
Deviation in fracture height of truncated cones between simulation and experiment.

The value of the forming limit curve for ductile fracture (FLDCRT) approaches 1.0 (indicating a damage condition), after which the mesh elements are removed. The corresponding maximum deformation height for each forming angle is provided in Table 7. The deviation between the simulated and experimental forming heights for varying forming angles is presented in Figure 17. Excellent agreement is observed, with the largest deviation at 4.6% for a forming tilt angle of 80 degrees.

This demonstrates the effectiveness of the proposed forming limit curve model, showing similarity in the forming height of the truncated cone samples during both simulation and experimentation. The results validate the reliability of the new FLCF model when predicting incremental sheet metal forming processes under different shaping angles.

## 6. Conclusions

This study delves into the critical role of the forming limit curve (FLC) in the numerical simulation of the single-point incremental forming (SPIF) process for Al1050 sheet material, followed by experimental validation. The investigation yields the following significant findings:

- Graphical method for FLC construction: a FLC is methodically constructed using the graphical approach and employed in numerical simulations to predict the variation in fracture height during SPIF. The results show a notable deviation between the simulated and experimental outcomes, particularly when forming the truncated cone with a 62° wall angle. The deviation, amounting to 15.97%, underscores the challenge in accurately modeling the SPIF process.
- Proposed forming limit curve at fracture in SPIF (FLCF): To enhance accuracy, a new FLCF is introduced based on the relationship between major and minor strains, drawing on the simulation results for truncated cone shaping with varying tool radii. The FLCF aligns remarkably well with experimental observations, especially for a 62° forming tilt angle, where the deviation is significantly reduced to 3.13%. This highlights the potential of the proposed FLCF in improving SPIF simulations.
- Validation across varied forming wall angles: To assess the reliability of the newly proposed FLCF, this study explores its effectiveness in predicting failure and forming fracture heights across a range of forming wall angles, spanning from 60° to 90°. The results are promising: for a 60° wall angle, the samples reach their maximum height without encountering fractures, with an FLDCRT value of 0.6292. With increasing forming wall angles (70°, 80°, 85°, 90°), the forming fracture height of the truncated cone tends to decrease, aligning closely with FLDCRT values approaching 1.0. The deviation in the forming fracture height between the simulations and experiments remains within acceptable limits, with the largest discrepancy observed at 4.6% in most cases.

In conclusion, this study not only highlights the necessity of an accurate FLC in SPIF simulations, but also introduces a novel FLCF model, which displays promising alignment with experimental results across various forming wall angles. This research contributes to the ongoing efforts to enhance the precision and reliability of the SPIF process, with potential applications in diverse industries requiring intricate sheet metal forming. 

## Figures and Tables

**Table 1:** Mechanical properties of Al1050 material.

Material	Al1050		
Rolling direction	0°	45°	90°
Yield strength (MPa)	81.6	79.7	89.8
Anisotropy coefficient (*r*)	0.62	0.38	0.85
Density (*ρ*, kg/mm^3^)	2.7 × 10^−6^		
Elastic modulus (*E*, kN/mm^2^)	69		
Poisson coefficient	0.33		

**Table 2:** Hill’48R’s material constants.

Value	*F*	*G*	*H*	*L*	*M*	*N*
Hill’48R	0.4503	0.6173	0.3827	1.5	1.5	0.9394

**Table 3:** Anisotropy coefficients of Hill’48R.

Coefficient	*R*_11_	*R*_22_	*R*_33_	*R*_12_	*R*_13_	*R*_23_
Hill’48R	1	1.0957	0.9679	1.2636	1	1

**Table 4:** Coefficients determined in two forming stages, based on Hill’48R stress function.

Coefficient		Tensile Strain (U.T)	Bi-Axial Tensile (B.T)
Hill’48R	α	0	1
	β	−0.5	1
	A(α)	1	0.597
	B(α)	0.213	0.171

**Table 5:** Major and minor strains with material models.

Coefficient		U.T	B.T
Kim–Tuan	$\bar{\varepsilon }$	0.415	0.625
	$\varepsilon_{1}$	0.410	0.354
	$\varepsilon_{2}$	−0.205	0.354

**Table 6:** Fracture heights and deviations between SPIF simulation and experiment.

Fracture Height	Experiment	Simulation	Deviation
Wall angle (^o^)	*h_e_* (mm)	$h_{s}$ (mm)	$\Delta h_{s}$ (%)
62	57.6	48.4	15.97%

**Table 7:** Deviation in fracture height between simulation and experiment for different wall angles.

Wall Angle (^o^)	60	70	80	85	90
Simulation, *h_s_* (mm)	131.2 (No failure)	19.8	18.2	16.9	15.7
Experiment, *h_e_* (mm)		19.5	17.4	16.3	15.2
$\mathrm{Deviation},\;\Delta h_{s}$		1.54%	4.60%	3.68%	3.29%
FLDCRT value	0.6292	1	1	1	1

## Data Availability

Data are contained within the article.
